# FGF8 isoform b expression in human prostate cancer

**DOI:** 10.1038/sj.bjc.6600875

**Published:** 2003-04-29

**Authors:** V J Gnanapragasam, M C Robinson, C Marsh, C N Robson, F C Hamdy, H Y Leung

**Affiliations:** 1 Prostate Research Group, School of Surgical Sciences, University of Newcastle upon Tyne, Framlington Place, Newcastle upon Tyne NE2 4HH, UK; 2 Department of Pathology, Freeman Hospital, Freeman Road, Newcastle upon Tyne, UK; 3 Academic Urology Unit, Division of Clinical Sciences, University of Sheffield, Royal Hallamshire Hospital, Glossop Road, Sheffield S10 2JF, UK

**Keywords:** FGF8b, prostate cancer, immunoreactivity, FISH

## Abstract

Overexpression of fibroblast growth factor 8 (FGF8) mRNA has been previously described in prostate cancer. Of its four isoforms, FGF8b is thought to be the most important in carcinogenesis. We hypothesised that immunodetection of FGF8b in archival prostate cancer specimens is of potential prognostic value. Using a selected cohort of prostate tumours from transurethral (*n*=30) and radical prostatectomies (*n*=59), an optimised protocol for FGF8b immunoreactivity was used to corroborate expression with clinical parameters. No expression was observed in benign prostates (*n*=10). In prostate cancer, immunoreactivity was localised to the malignant epithelium with weak signals in the adjacent stroma. Expression of FGF8b in stage T1 and T2 cancers were 40 and 67%, respectively. In contrast, FGF8b expression was present in 94% of T3 and 100% of T4 cancers. By histological grade, FGF8b was found in 41% of low-grade cancers (Gleason score 4–6), 60% of intermediate-grade cancers (Gleason score 7 and 92% of high-grade cancers (Gleason score 8–10). The intensity of expression was significantly associated with stage (*P*=0.0004) and grade (*P*<0.0001) of disease. We further hypothesised that FGF8b overexpression resulted from enhanced transcription and translation rather than from abnormalities involving the FGF8 gene locus. This was tested by means of fluorescent *in situ* hybridisation in 20 cancer specimens to map the FGF8 gene locus. FGF8 gene copy number in benign and malignant nuclei was found to be similar (2.33±0.57 and 2.0±0.81, respectively *P*=0.51). Based on these findings, we propose a multicentre study on cohorts of patients to further evaluate FGF8b as a potential prognostic marker in prostate cancer.

Prostate cancer is the second most common cause of cancer deaths in men in Western countries (Merril *et al*, 1996; Greenlee *et al*, 2000). For patients with locally advanced and/or metastatic disease, hormonal manipulation by androgen blockade is the mainstay of treatment. In hormone-relapsed disease however, there are no satisfactory therapeutic options. Targeted therapy of growth factors has been suggested as a model of treatment in both early and advanced cancers.

Human fibroblast growth factor 8 (FGF8) is a member of the fibroblast growth factor (FGF) family of 23 homologous proteins (Powers *et al*, 2000) and has been mapped to chromosome 10q24 (Yoshiura *et al*, 1997). The peptide was first implicated in prostate cancer when FGF8 mRNA was found to be expressed in prostate cancer cell lines (Ghosh *et al*, 1996; Schmitt *et al*, 1996). In clinical prostate cancer, overexpression of FGF8 mRNA was found to be associated with high-grade and late-stage disease (Leung *et al*, 1996; Dorkin *et al*, 1999). Subsequent work has shown that distinct isoforms of FGF8 possess different transforming potential. The human FGF8 gene comprises six exons (1A–D, 2 and 3) and alternate splicing in exon 1 produces four potential isoforms; a, b, e and f. Much attention has been focused on the b isoform because it exhibits potent *in vitro* transforming ability (MacArthur *et al*, 1995a; Ghosh *et al*, 1996). FGF8b transgenic mice have been shown to develop mammary and salivary gland tumours (Daphna-lken *et al*, 1998), while engineered overexpression of FGF8b in both prostate and breast cancer cell lines is known to induce a more aggressive phenotype displaying enhanced invasion and growth (Rudra-Ganguly *et al*, 1998; Ruohola *et al*, 2001).

We tested if an optimised protocol to quantitate FGF8b protein expression could be developed to study archival prostate cancer specimens. Using this method, we investigated the hypothesis that protein expression of the FGF8b isoform is closely associated with tumour grade and stage. Furthermore, we applied fluorescent *in situ* hybridisation (FISH) to study the significance of gene amplification or rearrangement as a mechanism for FGF8 overexpression.

## MATERIALS AND METHODS

### Antibody

Mouse monoclonal FGF8b antibody (500 *μ*g ml^−1^), obtained from R&D systems (Abingdon, UK), specifically recognises mouse and human FGF8b isoform. This antibody was produced from a mouse hybridoma in which the immunogen was an *Escherichia* *coli*-derived recombinant mouse FGF8b. We further confirmed specificity of the antibody by probing for recombinant FGF8b (rFGF8b – R&D systems) using Western blot analysis (data not shown).

### Patient samples

Samples were obtained from 59 patients undergoing radical prostatectomy (RP) with a median age of 62 years (range 38–71). None had received neo-adjuvant hormonal or chemotherapy treatment Whole-mount specimens were transected to reveal cancer foci and/or coincidental areas of high-grade prostatic intraepithelial neoplasia (HGPIN) and then embedded in paraffin wax blocks. A further 30 patients with prostate cancer (median age 72, range 56–82 years) diagnosed following transurethral resection (TUR) of the prostate were identified from a pathology department database. These patients initially presented with bladder outflow obstruction and none had prior hormone ablation therapy. Sections from a further 10 cases of benign prostatic hyperplasia (BPH) were also obtained from TURP archives and were confirmed to be histologically nonmalignant. Correlation of FGF8 expression in radical prostatectomy (RP) specimens were made to the pathological stage. Data obtained from TUR specimens were corroborated to their corresponding clinical stage. All cases of cancer were Gleason graded by a consultant uro-pathologist (MCR). Serum prostate-specific antigen (PSA) levels (first referral to the urology clinic or assay performed after surgery) were obtained from the patient's records.

### Immunohistochemistry

The optimal concentration of FGF8b antibody to be used for immunostaining was derived from serial dilution (1 : 100–1 : 1000) testing on composite tissue blocks. These blocks included samples of placenta as well as testis, breast cancer and prostate cancer known to express FGF8 (Tanaka *et al*, 1998; Dorkin *et al*, 1999). A dilution of 1 : 500 gave the optimal signals. As a negative control, sections of bronchus and ileum, which do not express FGF8, were stained with this concentration of antibody and failed to generate signals. Sections of prostate were cut at 4 *μ*m from tumour blocks and mounted onto APES-coated slides. Immunostaining was performed using a standard biotin/avidin–peroxidase method. Paraffin sections were baked overnight at 50°C, deparaffinised in xylene and rehydrated through graded alcohols with a final wash in distilled water. Antigen retrieval was achieved by immersion in 0.01 M sodium citrate buffer (pH 6.0) and microwaving on full power (1000 W) in a microwaveable pressure cooker to achieve 5 min at full pressure. Slides were then washed in running tap water and transferred to phosphate-buffered saline (PBS) pH 7.1 for 5 min. Treating sections for 10 min in 3% hydrogen peroxide quenched endogenous peroxidase activity. Slides were then rinsed in PBS and incubated with 20% normal goat serum in PBS for 10 min, followed by overnight incubation in optimally diluted primary antibody at 4°C. Slides were then incubated in secondary antibody (biotinylated goat anti-mouse, Biogenex) diluted 1 : 60 in PBS for 30 min at room temperature, rinsed in PBS and then treated with diaminobenzidine (Biogenex) solution for 5 min. Sections incubated without any primary antibody were also employed as internal negative controls. All sections were counterstained with haematoxylin.

### Fluorescent probe manufacture

A human BAC clone from the 10q24 region (bA573E23) (Acc no: AC016774-clone RP11-573E23) was kindly provided by Dr Mark Earthrowl (Sanger Centre-Chromosome 10 mapping group, UK). This BAC clone was selected based on a 100% similarity match between the FGF8 cDNA sequence and known sequences within the clone (BLAST (NCBI) program). The clone was colony polymerase chain reaction (PCR) verified by the distributor and found to contain the expected markers. Stabs were streaked onto chloramphenicol (20 *μ*g ml^−1^) plates and grown overnight in a 37°C incubator. Following standard extraction, BAC DNA at a concentration of 1 *μ*g *μ*l^−1^ was labelled using Nick Translation according to the manufacturer's protocol (Vysis Inc., IL, USA). In this procedure, Spectrum Green dUTP was incorporated into the probe fragments generated. Temperature and timing of the labelling reaction was set to produce probes of size between 200 and 300 bp. As a competitive step, 2 *μ*l of salmon sperm DNA (Sigma, Poole, UK) was added to 10 *μ*l of probe and ethanol precipitated. Pelleted probe was then resuspended in Hybrisol VI (Oncor, UK) to a concentration of 10 ng *μ*l^−1^. A prelabelled *α* satellite probe specific for chromosome 10 (centromere) was obtained from Oncor, UK.

### Fluorescent *in situ* hybridisation (FISH)

Paraffin tissue sections of prostate cancer (*n*=20) and benign prostatic hyperplasia (*n*=10) were predried at 50°C overnight before being dewaxed in xylene and rehydrated in ethanol. To improve signal intensity, sections were incubated in 1 M sodium thiosulphate (pH 7.0) for 10 min at 75°C. Pepsin (0.4%) in 0.2 M HCl (Sigma, Poole, UK) was used to reverse protein crosslinks caused by formalin fixation for a period of 26 min. Sections were denatured in 70% formamide (in 2 × SSC, pH 7.0) at 75°C for 2 min before quenching in ice-cold ethanol. Preprepared probe was denatured at 75°C for 5 min and 200 ng of probe was added to each section and the slide incubated at 37°C overnight in a humidified chamber. Sections were washed in 2 × SSC, then 4 × SSC plus Triton before being counterstained with DAPI (4,6 diamidino-2 phenylindole-2 hydrochloride) in antifade mounting medium (Vectashield, Vectorlabs, Peterborough, UK).

### Slide evaluation

Immunostained sections were studied under a light microscope without prior knowledge of the clinical details. Sample sections were first previewed by two independent observers (VJG, MCR) and interobserver agreement was obtained regarding a grading system. The level of FGF8b expression was correlated directly with the strength of the immunoreactivity signal generated and scored as either absent (−), weak (+), moderate (++) or strong (+++). This template was then used for the series as a whole. Where two or more signal intensities were present in one case, the intensity with greater than 50% area of staining was taken as the score. Differences in malignant epithelial expression of FGF8b protein in relation to clinical parameters were then examined using the Kruskall–Wallis test. A *P*-value of <0.05 was taken to indicate statistical significance and analysis was performed using the Arcus QuickStat programme. Clinical parameters studied were pathological or clinical stage, histological grade and serum prostate-specific antigen (PSA) level. Stage was defined according to the TNM classification system whereby T1–T2 was classed as organ confined and T3–T4 as locally advanced. Gleason sum score was used to assess grade and is the sum of the two predominant grades (range 1–5) of cancer present in each individual case.

For FISH analysis, signals were visualised using a Digital Scientific SmartCapture Workstation (Cambridge, UK). Probe authenticity was first verified on blood lymphocyte metaphase spreads. Mean chromosome copy number (total number of hybridisation signals divided by the total number of nuclei) was used as a measure of the overall chromosome copy number in paraffin sections. In both benign and cancer cases, this was determined by probing matched histological sections with a chromosome 10 centromeric probe. The mean chromosome counts from at least three tumour areas were used to give an overall value for each case. For FISH in paraffin sections, copy number was assessed by counting the number of signals/nuclei in at least 20 signal-positive nonoverlapping cells in both benign and malignant tissues. At least three separate fields from each tissue section were analysed separately.

## RESULTS

### FGF8b expression and clinical parameters

Among radical prostatectomies (RP), the majority of cases were organ confined (50 out of 59) and the remaining nine had extracapsular extension (pT3) (Table 1

**Table 1:** FGF8b staining in radical prostatectomies and transurethral resections

	**FGF8b expression**					
**Clinical details**	**–**	**+**	**++**	**+++**	**Total**	***P-*value**
BPH	10	0	0	0	10	—
Prostate cancer						
*Stage*						
RP (*n*=59)						
T1	19	4	2	0	25	
T2	10	5	8	2	25	
T3	1	4	3	1	9	
T4	0	0	0	0	0	0.01
TUR (*n*=30)						
T1	2	0	6	2	10	
T2	1	2	4	2	9	
T3	0	1	4	3	8	
T4	0	0	1	2	3	0.34
*Gleason grade*						
RP (*n*=59)						
4–6	16	7	3	0	26	
7	12	5	7	2	26	
8–10	2	0	3	2	7	0.03
TUR (*n*=30)						
4–6	2	0	3	0	5	
7	1	0	3	3	7	
8–10	0	3	9	6	18	0.11

Stage is defined according to the TNM classification system for prostate cancer. Histological grade was defined by the Gleason score, being the sum of the two predominant grades of cancer in a section (range 2–10). Grades was then defined as low (Gleason score 4–6), moderate (Gleason score 7) or high (Gleason score 8–10). A *P*-value of less than 0.05 was taken to indicate statistical significance. RP=radical prostatectomy; TUR=transurethral resection.

). In this group, 26 out of 59 cases were low grade (Gleason sum score 4–6), 26 out of 59 were intermediate grade (Gleason sum score 7) while seven were high grade (Gleason sum score 8–10). Among transurethral resected (TUR) specimens, the case mix was different. On clinical staging, 19 out of 30 were organ confined and 11 out of 30 locally advanced. By Gleason sum score, five were low grade, seven intermediate and 18 high grade (Table 1). This latter group therefore had a tendency towards locally advanced and higher-grade disease. FGF8b immunoreactivity was found to be associated with disease stage and grade in the RP group (Table 1). This group however had few patients with advanced stage and/or grade cancers and concomitantly the level of significance was not high (*P*=0.01 and 0.03, respectively). In the TUR group, FGF8b expression was not associated with either clinical parameter most likely because of a preponderance of advanced-stage and/or high-grade disease (Table 1). To obtain a better mix of cases, the RP and TUR cohorts were combined and then analysed.

FGF8b-negative cases were found predominantly in organ-confined and low- or intermediate-grade cancers (Table 2

**Table 2:** Intensity of FGF8b expression is associated with stage and grade of prostate cancer

	**FGF8b expression**					
**Prostate cancer (RP and TUR)**	**–**	**+**	**++**	**+++**	**Total**	***P*-value**
*Stage* (*n*=*89*)						
T1	21	4	8	2	35	
T2	11	7	12	4	34	
T3	1	5	7	4	17	
T4	0	0	1	2	3	0.0004
Gleason grade (*n*=89)						
4–6	18	7	6	0	31	
7	13	5	10	5	33	
8–10	2	3	12	8	25	<0.0001
PSA (*n*=72)	10.2	19.1	35.7	34.1		0.29
Mean value and range (ng ml^−1^)	(0.8–25.9)	(1.7–154)	(1.7–200)	(2.8–161)		

Data from the radical prostatectomy (RP) and transurethral cohorts were combined to analyse the association of FGF8 with clinical parameters. PSA values in each signal intensity group were also analysed. A *P*-value of less than 0.05 was taken to indicate statistical significance. RP=radical prostatectomy; TUR=transurethral resection.

). Stage T1 and T2 cancers expressed FGF8b in 40% (14 out of 35) and 67% (23 out of 34) of cases, respectively. In T3 and T4 disease, FGF8b expression was detected in 94% (16 out of 17) and 100% (3 out of 3) of cases, respectively (Table 2). This increase in prevalence was associated with an increase in signal intensity and FGF8b immunoreactivity was significantly associated with advanced-stage disease (*P*=0.0004) (Table 2). By histological grade, 41% (13 out of 31) of low-grade cancers (Gleason score 4–6) expressed FGF8b, while 60% (20 out of 33) of intermediate-grade cancers (Gleason score 7) expressed FGF8b (Table 2). High-grade cancers (Gleason score 8–10) expressed FGF8b in 92% (23 out of 25) of cases. The level of FGF8b immunoreactivity was significantly associated with increasing grade of cancer (*P*<0.0001). Serum PSA levels were available in 72 out of 89 cases. Analysis of the mean and range of values in each signal intensity group demonstrated that serum PSA was not associated with the level of expression of FGF8b (Table 2).

In summary, these results demonstrate that FGF8b expression is both stage and grade associated. Where FGF8b was not expressed, cancers tended to be organ confined and low grade. As cancer progressed to more advanced and high-grade disease, FGF8b was found to be more prevalent and expression concomitantly upregulated.

### Expression pattern of FGF8b

**Figure 1:**
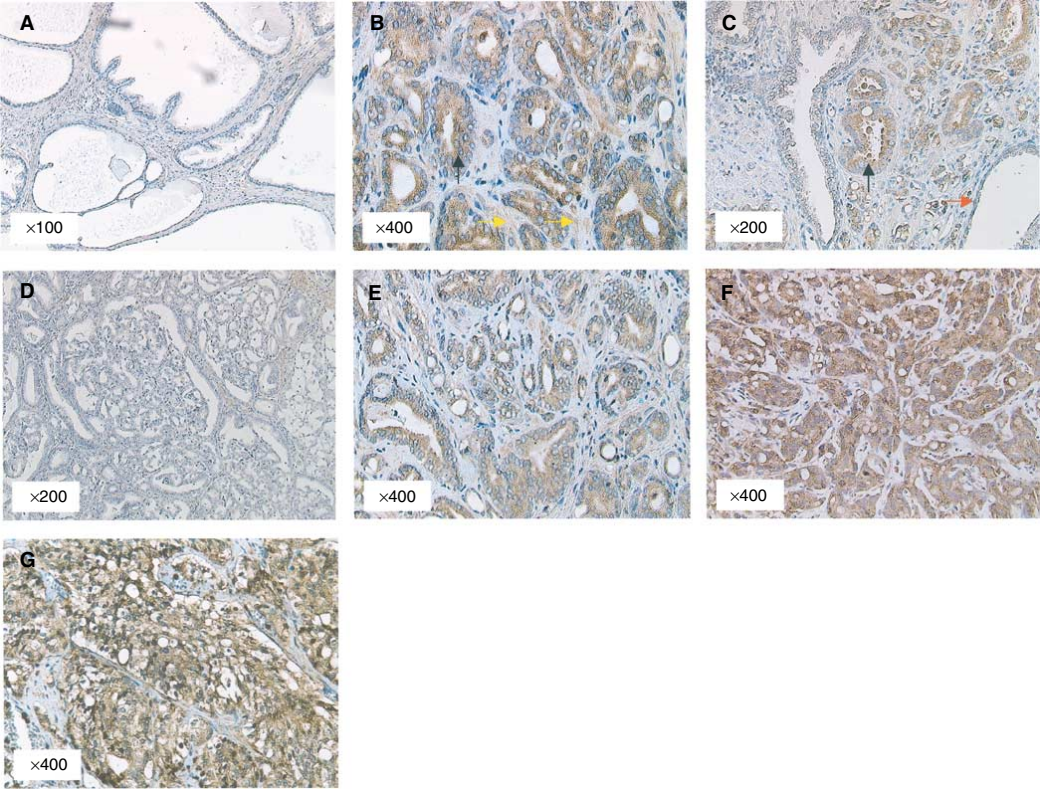
FGF8b expression pattern in prostate cancer. (**A**) Benign prostate with no staining for FGF8b. (**B**) Malignant foci (black arrow) of prostate cancer demonstrating FGF8b positivity in epithelial cells and associated weak stromal signals (yellow arrow). (**C**) Sections of malignant epithelium adjacent to benign glands (red arrow). (**D**) FGF8b-negative high-grade prostatic intraepithelial neoplasia from a radical prostatectomy. (**E**–**G**) Sections of prostate demonstrating low, moderate and high FGF8b expression, respectively, in different histological grades of cancer.

In samples of benign prostatic hyperplasia (*n*=10), no FGF8b expression was detected (Figure 1A

). In radical prostatectomies, FGF8b was found predominantly in the epithelium of malignant glands (Figure 1B, C, E). In these cells, we observed that FGF8b was located primarily in the cytoplasm of epithelial cells with no expression seen in the nucleus. In the majority of cases, we also observed patchy signals in the stroma adjacent to FGF8b-expressing epithelial cells (Figure 1B). This observation is in keeping with FGF8b being a secreted paracrine peptide. Areas of normal prostate epithelium in the same slide were invariably negative for FGF8b expression (Figure 1C). Coincidental areas of high-grade prostatic intraepithelial neoplasia (HGPIN) were found adjacent to tumour in 10 radical prostatectomies. Among these, five cases were negative for FGF8b in both cancer and HGPIN areas (Figure 1D) and two cases were positive for FGF8b in both cancer and HGPIN areas. In a further two cases, areas of cancer expressed FGF8b but coexisting HGPIN did not. There was only one case in which HGPIN was found to express FGF8b but coexisting cancer was negative. Among patients diagnosed following TUR, the majority of tumours were of high-grade cancer. FGF8b expression was again found predominantly in malignant epithelium (Figure 1F, G).

### FGF8 gene locus is not amplified in malignant cells

**Figure 2:**
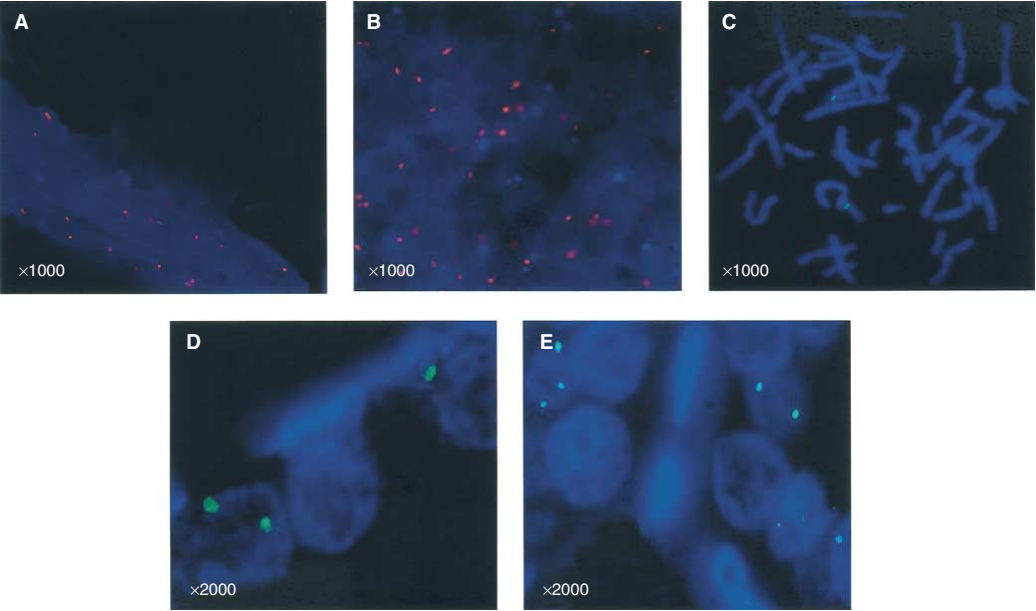
FGF8 gene locus analysis. Nuclei and chromosomes are stained blue by DAPI as in Materials and Methods. (**A**, **B**) Fluorescent *in situ* hybridisation using a chromosome 10 specific probe (red fluorescent) for analysis of the mean chromosome copy number in benign and malignant prostate sections, respectively. (**C**) Specific localisation of the green fluorescent-labelled BAC probe to the short arm of chromosome 10 in blood metaphase spreads. (**D**) Paraffin FISH using fluorescent-labelled BAC probe in a benign prostate section. (**E**) Paraffin FISH using fluorescent-labelled BAC probe in prostate cancer.

The mean chromosome copy number (MCCN) for benign tissue was 2.0±0.81 and for malignant tissue 1.60±0.54, suggesting that there was no statistical difference in chromosome 10 copies between benign and malignant nuclei (Figure 2A, B

). Previous FISH studies have mapped the FGF8 gene to a region within the 10q24 band using blood lymphocyte metaphase spreads (Yoshiura *et al*, 1997). Our BAC probe containing the FGF8 sequence was similarly tested on blood lymphocyte metaphase spreads and found to hybridise specifically to the 10q24 region (Figure 2C). Probes were then hybridised to optimally prepared paraffin-embedded prostate sections. In signal-positive nuclei, there was no observable difference in the number of hybridisation signals between benign and malignant cells. The mean number of signals in benign nuclei was 2.33±0.57 (Figure 2D) and for malignant nuclei 2.0±0.81 (*P*=0.51) (Figure 2E). This suggests that the region covered by the FISH probe, and by inference its constituent genes including FGF8, is neither amplified nor displays major rearrangement during prostate carcinogenesis.

## DISCUSSION

FGFs exert their biological function by binding to high-affinity tyrosine kinase fibroblast growth factor receptors (FGFR) in the presence of heparin (Ornitz *et al*, 1992). Four receptors have been detailed (designated FGFR 1–4) and multiple splice variants of these receptors have been described (Ornitz *et al*, 1996). FGF8 is known to activate FGFR2, 3 and 4, as well as FGFR1 if applied in high concentrations (Ornitz *et al*, 1996; Blunt *et al*, 1997). In addition, developmental studies have shown that it is the c splice forms of FGFRs that are the preferred targets of FGF8 and these are expressed predominantly in the mesenchymal tissue (MacArthur *et al*, 1995b). This suggests that FGF8, commonly secreted by epithelial cells, interacts with stromal tissue in a paracrine fashion. The prostate is known to express FGFR1, 2 and 3, while FGFR4 has only been detected at low levels in prostate cancer cell lines (Story *et al*, 1994; Ittman and Mansukhani, 1997; Giri *et al*, 1999; Ropiquet *et al*, 2000). In prostate carcinogenesis, FGFR splicing characteristics are known to be altered. In early malignancy, for instance, the FGFR1IIIc isoform is preferentially expressed by epithelial cells while in more advanced cancers, the FGFR2IIIc isoform is upregulated (Yan *et al*, 1992; Kwabi-Addo *et al*, 2001). In this context, FGF8 may well adopt both a paracrine and an autocrine role. The FGF8 isoforms themselves are known to have relatively specific binding characteristics (Blunt *et al*, 1997). Human FGF8a, for example, does not activate any of the FGFRs, while the FGF8b and e isoforms are known to interact with FGFR3IIIc and FGFR4 (Blunt *et al*, 1997). FGF8b however is the only isoform known to interact with FGFR2IIIc. It is clear that a more favourable environment for FGF8, and in particular FGF8b appears to be acquired in cancer progression. This idea is further supported by evidence of transcript overexpression in metastatic lesions of prostate cancer (Dorkin *et al*, 2000).

Previous expression studies, including work from our own centre, have assayed total FGF8 levels in clinical material (Leung *et al*, 1996; Dorkin *et al*, 1999; Valve *et al*, 2001). Data concerning FGF8 correlation with clinical parameters, however, have been conflicting and may reflect differences in splice form utilisation (Leung *et al*, 1996; Tanaka *et al*, 1998; Dorkin *et al*, 1999; Valve *et al*, 2001). Transcript studies of isoform expression are limited by reliance on semiquantitative RT–PCR on heterogeneous frozen materials. To allow quantitative and spatial assessment, we optimised a protocol for immunohistochemical detection of FGF8b. The choice of antibody was crucial for this study. We have previously described the use of the selected FGF8b antibody in both Western blot and immunohistochemistry studies (Gnanapragasam *et al*, 2002). The specificity and suitability of this antibody for immunohistochemistry has also been previously validated in breast cancer (Zammit *et al*, 2002). Furthermore, the antibody was shown not to crossreact with other FGFs. In this study, specificity of the antibody was confirmed and immunoreactivity was blocked completely by preabsorption with recombinant FGF8b.

In prostate cancer specimens, we found that FGF8b was expressed in malignant epithelium and to a lesser extent in the adjacent stroma. It is possible that FGF8b, secreted by epithelial cells, stimulates stromal cells to manufacture and secrete other growth factors and cytokines, which in turn stimulate epithelial cells. This theory is supported by FGFR1IIIc and FGFR3IIIc splice form expression in the prostatic stroma (Story *et al*, 1994). Immunohistochemistry demonstrated that FGF8b protein was significantly overexpressed in locally advanced and high-grade tumours. Tanaka *et al* (1998) have similarly reported frequent overexpression of FGF8b protein in malignant prostate epithelium. In a study of 43 needle biopsies of prostate cancer however, the authors did not observe an association between staining intensity and clinical parameters. This study however did not use the Gleason grade and TNM system of disease classification but a Japanese system of prostate cancer classification. Valve *et al* (2001), investigating isoform transcript expression, did not find a difference in FGF8b levels comparing normal and malignant prostates using semiquantitative PCR. To explain this, the authors suggest that FGF8b mRNA in their normal prostates might have been derived from foci of incidental prostatic intraepithelial neoplasia (PIN). We similarly observed that in prostate cancer sections, some coincidental areas of HGPIN demonstrated positivity for FGF8b. Serum PSA levels were not found to be associated with FGF8b immunoreactivity.

Loss of heterozygosity (LOH) on the short arm of chromosome 10 has been documented in both familial and sporadic prostate cancer as well as in HGPIN (Saric *et al*, 1999; Verhagen *et al*, 2000; Sakr and Partin, 2001). Most recently, Latini *et al* (2001), using microsatellite markers, have shown LOH of the candidate tumour suppressor PTEN gene locus (10q23) in advanced prostate cancer. Using a labelled probe spanning the FGF8 gene locus, FISH analysis in a series of 20 cancers failed to show evidence of a change, neither amplification nor rearrangement, within this region. As both FGF8 mRNA and protein levels are increased in prostate cancer, this points to transcriptional control of the gene as a mechanism of regulating expression. In keeping with this, we have recently demonstrated that the androgen receptor is able to enhance FGF8 gene promoter activity (Gnanapragasam *et al*, 2002).

The mechanism by which FGF8 mediates tumorigenesis has been the subject of much research. Ghosh *et al* (1996) were the first to demonstrate that human FGF8b induced marked morphological transformation in NIH3T3 fibroblast cells and strong tumorigenicity of the transfected cells in nude mice. More recently, FGF8b has been overexpressed in LNCaP prostate cancer cells by infection with an engineered retroviral construct (Song *et al*, 2000). Overexpression of FGF8b doubled LNCaP cell growth and enhanced anchorage-independent clonogenicity. In addition, while noninfected LNCaP cells failed to form tumours in nude mice, the majority of transfected cells (four out of five inoculations) established multifocal growths. In this context, FGF8b appeared to function as an autocrine stimulator of growth and proliferation. The authors also demonstrated that FGF8b-transfected LNCaP cells were more efficient in migrating through Matrigel basement membranes. They postulated that this was because of enhanced secretion of enzymes capable of digesting the extracellular matrix. In support of this, Ruohola *et al* (2001) found that MCF7 breast cancer cells transfected with FGF8b were similarly more invasive than controls and upregulated the expression of matrix metalloproteinase 9. Tumours produced by FGF8b-expressing MCF7 cells were also shown to be more vascular than untransfected controls (Ruohola *et al*, 2001). Mattila *et al* (2001) have also demonstrated that FGF8b is able to stimulate migration and sprouting of mouse brain capillary endothelial cells. In this study, FGF8b promoted endothelial cell differentiation, organisation of capillary cells into cords and formation of lumen-like structures. From these data, it is reasonable to postulate that expression of high levels of FGF8b is conducive to the induction of a more aggressive malignant phenotype. Such cancer cells may then display enhanced growth and invasion as well as have angiogenic-inducing properties.

In conclusion, we demonstrate in this study the first evidence of an association between FGF8 isoform b protein expression and clinical prostate cancer. We have shown that the expression of the b isoform of FGF8 is limited to malignant prostate epithelium, the adjacent stroma and to occasional areas of HGPIN. Expression is significantly upregulated in late-stage and high-grade disease, and appears to occur at the transcriptional level as the gene locus is not amplified. Taken together, these results show evidence of a role for FGF8b in mediating prostate cancer progression, and in this context we believe that FGF8b may have an important role as a prognostic factor in disease outcome. We now propose a multicentre study to further corroborate our findings and to evaluate the role of FGF8b immunodetection as a prognostic marker in patients with prostate cancer.
